# Buildup from birth onward of short telomeres in human hematopoietic cells

**DOI:** 10.1111/acel.13844

**Published:** 2023-04-28

**Authors:** Tsung‐Po Lai, Simon Verhulst, Sharon A. Savage, Shahinaz M. Gadalla, Athanase Benetos, Simon Toupance, Pam Factor‐Litvak, Ezra Susser, Abraham Aviv

**Affiliations:** ^1^ Center of Human Development and Aging, New Jersey Medical School, Rutgers The State University of New Jersey Newark New Jersey USA; ^2^ Groningen Institute for Evolutionary Life Sciences University of Groningen Groningen The Netherlands; ^3^ Division of Cancer Epidemiology and Genetics National Cancer Institute Bethesda Maryland USA; ^4^ INSERM DCAC Université de Lorraine Nancy France; ^5^ CHRU‐Nancy Pôle Maladies du vieillissement Gérontologie et Soins Palliatifs and Fédération Hospitalo‐Universitaire CARTAGE‐PROFILES Université de Lorraine Nancy France; ^6^ Department of Epidemiology Mailman School of Public Health New York New York USA; ^7^ Department of Psychiatry New York State Psychiatric Institute New York New York USA

**Keywords:** age, lifetime, sex, Southern blotting, subtelomeric region, telomere biology disorders, telomeres, terminal restriction fragments, TeSLA

## Abstract

Telomere length (TL) limits somatic cell replication. However, the shortest among the telomeres in each nucleus, not mean TL, is thought to induce replicative senescence. Researchers have relied on Southern blotting (SB), and techniques calibrated by SB, for precise measurements of TL in epidemiological studies. However, SB provides little information on the shortest telomeres among the 92 telomeres in the nucleus of human somatic cells. Therefore, little is known about the accumulation of short telomeres with age, or whether it limits the human lifespan. To fill this knowledge void, we used the Telomere‐Shortest‐Length‐Assay (TeSLA), a method that tallies and measures single telomeres of all chromosomes. We charted the age‐dependent buildup of short telomeres (<3 kb) in human hematopoietic cells from 334 individuals (birth‐89 years) from the general population, and 18 patients with dyskeratosis congenita‐telomere biology disorders (DC/TBDs), whose hematopoietic cells have presumably reached or are close to their replicative limit. For comparison, we also measured TL with SB. We found that in hematopoietic cells, the buildup of short telomeres occurs in parallel with the shortening with age of mean TL. However, the proportion of short telomeres was lower in octogenarians from the general population than in patients with DC/TBDs. At any age, mean TL was longer and the proportion of short telomeres lower in females than in males. We conclude that though converging to the TL‐mediated replicative limit, hematopoietic cell telomeres are unlikely to reach this limit during the lifespan of most contemporary humans.

## Graphical abstract

Population studies have principally focused on the role of mean telomere length in human health and longevity. The availability of new telomere length measurement techniques enables examining the relations between the shortest telomeres and human diseases.

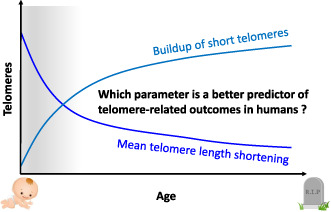

**Abbreviations**
bp: base pairs
DC/TBD: dyskeratosis congenita‐related telomere biology disorders
FISH: fluorescent in situ hybridization
HC: hematopoietic cell
kb: kilobase
LTL: leukocyte telomere length
qPCR: quantitative polymerase chain reaction
S: single gene product
SB: Southern blotting
SBmTL: mean TL using SB
SNP: single‐nucleotide polymorphism
T: amplified telomere product
TeS3kb: proportion of telomeres less than 3 kb, measured by TeSLA
TeSmTL: mean TL using TeSLA
TL: telomere length
TRFs: terminal restriction fragments

## 1 INTRODUCTION

Telomere length (TL) is highly variable across adult humans (Aviv & Shay, 2018). Several lines of evidence indicate, however, that this variation, as expressed in leukocyte TL (LTL), is principally established at birth. First, LTL variation (SD ~ 700 base‐pairs (bp)) among newborns is like that among their parents (Factor‐Litvak et al., 2016) and adults in the general population (Aubert et al., 2012; Steenstrup et al., 2017). Second, heritability, observed from birth onward, explains a major component of the LTL variation among individuals (Factor‐Litvak et al., 2016; Hjelmborg et al., 2015; Slagboom et al., 1994). Third, LTL tracks after the first decade of life, such that individuals maintain their relative LTL ranking, for example, short, or long LTL, compared to their peers (Benetos et al., 2013, 2019). Fourth, exposures and behaviors exert a nominal effect on LTL in adults (Bountziouka et al., 2022; Pepper et al., 2018). Having short or long LTL thus precedes by decades the clinical onset of aging‐related diseases. Jointly, this LTL precedence and Mendelian randomization analyses of SNPs associated with LTL infer a causal role of TL in many aging‐related diseases and longevity in humans (Codd et al., 2021). It follows that LTL at birth and progressive telomere shortening afterward might contribute to aging‐related diseases (Aviv & Shay, 2018; Shay, 2016) and longevity (Arbeev et al., 2020; Codd et al., 2021) in humans. Data of change with age in LTL parameters are thus vital for understanding the role of telomeres in human health and longevity.

Epidemiologists and geneticists have relied on several TL measurement techniques in population studies. These include quantitative polymerase chain reaction (qPCR) (Cawthon, 2002, 2009), Southern blotting (SB) of the terminal restriction fragments (TRFs) (Kimura, Stone, et al., 2010), and flow‐fluorescent in situ hybridization (FISH) (Baerlocher et al., 2006; Rufer et al., 1998). Such techniques measure the mean length of the 92 telomeres at the ends of the p and q arms of the 23 pairs of human chromosomes. However, the shortest telomeres, rather than mean length of all these telomeres, trigger cessation of somatic cell replication (Hemann et al., 2001; Zou et al., 2004).

LTL dynamics (LTL at birth and its age‐dependent shortening) have been charted based on measurements of mean TL (Aubert et al., 2012; Steenstrup et al., 2017). However, the buildup of short/ultrashort telomeres in hematopoietic cells (HCs) over the human life course might be a better indicator of TL‐dependent mechanisms that contribute to aging‐related diseases. For technical reasons (Materials and Methods), however, SB, considered the “gold standard” of TL measurements, provides limited information about telomeres shorter than (<) 3 kb. A recently developed method, the Telomere‐Shortest‐Length‐Assay (TeSLA), tallies and measures single telomeres (Lai et al., 2017), and provides a comprehensive picture of their distribution that includes telomeres with length of a few 100 bps. These features may explain the ability of TeSLA to generate further understanding of the potential role of telomeres in human biology and disease (Benetos et al., 2021; Lai et al., 2021).

## 2 RESULTS

We first describe features of SB and TeSLA, and then present data that chart for the first time a progressive buildup of telomeres <3 kb in HCs across nine decades of the human lifespan.

### 2.1 Features of SB and TeSLA

SB (Kimura, Stone, et al., 2010) and TeSLA (Lai et al., 2017) measure the lengths of the TRFs. TeSLA, however, detects and measures, in addition, TRFs that are <3 kb (Materials and Methods, illustrated in Figure S1).

**FIGURE 1:**
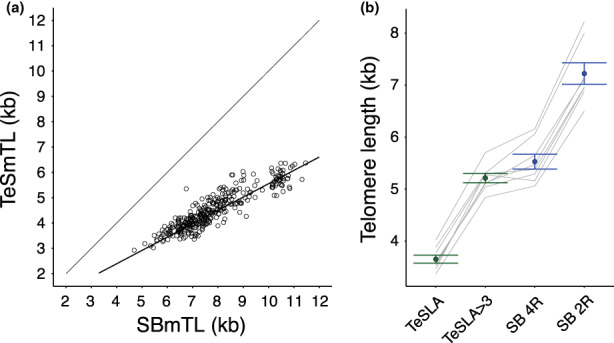
The relation between mean TL, measured by SB versus mean TL, measured by TeSLA. TeSmTL (kb) in relation to SBmTL (kb). N = 334. Bold Line is the regression line (Table 2). Also shown is line of unity (Y = X). Right panel. Mean (±SE) TL (kb) was estimated in four ways. Light grey lines connect different methods applied to the same samples (*n* = 8). Methods: TeSLA: TeSmTL; TeSLA 3 kb, TeSmTL but calculated excluding telomeres <3 kb; SB 4R: SBmTL but DNA digested using the 4 restriction enzymes used in TeSLA; SB 2R: SB with DNA digested using the 2 restriction enzymes routinely applied. For comparability, both TRF estimates were calculated over the same range (3–20 kb).

We measured TL parameters in leukocytes from a cohort of 334 individuals (birth‐89 years) originating from the general population (Table 1). Mean TL, measured using TeSLA (TeSmTL), strongly correlated with, but was consistently shorter than mean TL, measured with SB (SBmTL) (Table 2; Figure 1, left panel). This difference stems in part from the absence of telomeres <3 kb in the SB output. Excluding telomeres <3 kb from the TeSLA output thus raised the TeSmTL from 4.51 ± 0.77 kilo‐base (kb) (SD) to 5.82 ± 0.63 kb, a value that was still ~2 kb shorter than SBmTL (7.83 ± 1.40 kb) for the entire cohort. The TRFs include the sub‐telomeric region (Kimura, Stone, et al., 2010), a segment proximal to the canonical telomeres (TTAGGG tandem repeats), known as the X region (Steinert et al., 2004). The length of the X region largely depends on the restriction enzymes used to digest the DNA (Kimura, Stone, et al., 2010), and TeSLA employs different restriction enzymes than SB (Lai et al., 2017). For a subsample (*n* = 8), we generated, therefore, TRFs for SB by the same set of restriction enzymes used for TeSLA. This resulted in 1.69 ± 0.22 kb (mean ± SD) shorter SBmTL (7.22 ± 0.59 kb vs. 5.53 ± 0.40 kb; Figure 1, right panel). TeSmTL of this subset with and without telomeres <3 kb were 3.65 ± 0.22 kb and 5.21 ± 0.25 kb, respectively. Thus, two effects, that is, telomeres <3 kb and the different restriction enzymes used to generate the TRFs, explain 92% of the difference between TeSmTL and SBmTL. Still, an unexplained difference of 0.31 ± 0.27 kb remains between TeSmTL and SBmTL (paired‐*t*‐test, *t* = 3.28, *p* = 0.01), suggesting we either slightly underestimated TeSmTL, or slightly overestimated SBmTL. Further research is warranted therefore to understand the unexplained 8% of the difference between the estimates.

**TABLE 1:** Summary of cohorts from the general population.

Cohort	*N* (Female/Male)	Age range	Reference
1	59 (30/29)	0–40	2
2	136 (79/57)	6–74	7
3	20 (14/6)	75–89	32
4	109 (54/55)	15–74	‐^a^
5	10 (0/10)	45–61	33

^a^ Donors of hematopoietic cell transplant in an ongoing study.

**TABLE 2:** Mean telomere length measured using TeSLA (TeSmTL) in relation to telomere length measured using Southern blot (SBmTL).

	Estimate (SE)	*t*	*p*
Intercept	0.246 (0.194)	1.27	0.22
SBmTL (kb)	0.551 (0.022)	24.42	<0.0001

*Note*: Residual variance: 0.087; variance explained by cohort: 0.066; *R* ^2^ = 0.78.

*Abbreviation*: SE, standard error.

### 2.2 Cross‐sectional data of leukocyte TL parameters over nine decades of life

**FIGURE 2:**
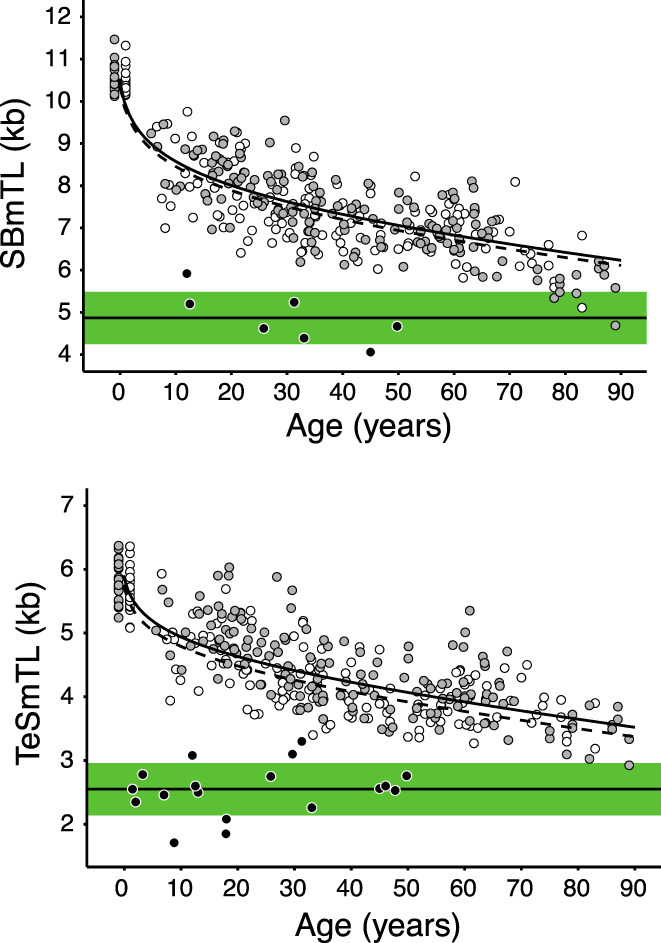
Mean TL versus age. Upper panel. SBmTL, mean TL by SB. Bottom panel. TeSmTL, mean TL by TeSLA. Females: open markers, continuous line. Males: grey markers, dashed line. Dyskeratosis congenita patients: solid black markers, mean ± 1 SD indicated by the green shaded area. Newborns' data are slightly displaced left and right of age zero to reduce the number of overlapping data points.

SBmTL in the cohort showed a steep and rapidly decelerating pace of telomere shortening during childhood, but relatively steady pace during adulthood. (Figure 2, top panel). This trajectory of SBmTL was best described by a model that included the natural logarithm of age (+1) together with age (Table 3). In this model, the linear age term slightly tilted the equation to fit the regression to the long SBmTL of newborns. As per earlier findings, LTL was longer in females compared to males (Aubert et al., 2012; Factor‐Litvak et al., 2016; Gardner et al., 2014; Hjelmborg et al., 2015; Hunt et al., 2020; Steenstrup et al., 2017) (Table 3, and below). We next compared SBmTL in our sample with SBmTL in seven patients with dyskeratosis congenita‐related telomere biology disorders (DC/TBDs) (Alter et al., 2012; Niewisch et al., 2022). In previous work we defined the TL‐mediated replicative limit in human HCs as the “telomeric brink” based on SBmTL in these patients (Steenstrup et al., 2017). In general, SBmTL in the oldest participants from the general population showed little overlap with the SBmTL in DC/TBDs (Figure 2, top panel).

**TABLE 3:** Telomere metrics in relation to age and sex.

	Estimate (SE)	T	*p*
(A) SBmTL			
Intercept	10.405 (0.191)	54.36	<0.0001
Ln (age + 1)	−0.720 (0.073)	9.79	<0.0001
Age	−0.010 (0.003)	3.06	0.002
Sex (male)	−0.121 (0.065)	1.87	0.063
(B) TeSmTL			
Intercept	5.481 (0.118)	49.43	<0.0001
Ln (age + 1)	−0.340 (0.049)	6.94	<0.0001
Age	−0.0087 (0.0023)	3.86	0.0002
Sex (male)	−0.149 (0.045)	3.32	0.001
(C) TeS3kb			
Intercept	0.476 (0.0179)	26.53	<0.0001
Ln (age + 1)	0.0260 (0.0078)	3.35	<0.002
Age	0.00199 (0.00035)	5.82	<0.0001
Sex (male)	0.0256 (0.0069)	3.71	<0.00025

*Note*: (A): SBmTL: mean LTL, measured by Southern blot; *R* ^2^ = 0.80; Variance explained by cohort: 0.074, residual variance: 0.337. (B): TeSTL: mean LTL, measured by TeSLA; *R* ^2^ = 0.70; Variance explained by cohort: 0.022, residual variance: 0.161. (C): TeS3kb: Proportion of telomeres <3.0 kb, measured by teSLA, was arcsine square root transformed. *R* ^2^ = 0.59; Variance explained by cohort: 0.0005, residual variance: 0.0038.

TeSmTL declined similarly with age as SBmTL (Figure 2, bottom panel). It was also best described by a model that included the natural logarithm of age (+1) together with age and sex (Table 3). TeSmTL in our sample showed little overlap with TeSmTL in 18 patients with DC/TBDs (Figure 2, bottom panel).

**FIGURE 3:**
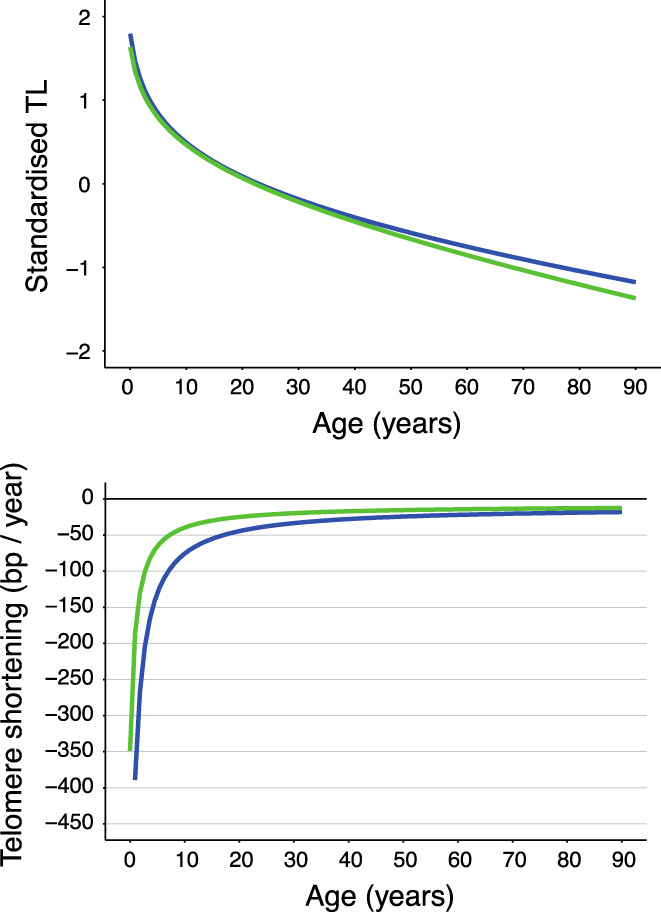
Comparison of association with age between mean TL by TeSLA, with mean TL by SB. TeSmTL, mean TL by TeSLA; SBmTL, mean TL by SB. Upper panel. TL measurements with each method was transformed to standard normal distribution (mean = 0, SD = 1). Lower Panel. Telomere shortening in relation to age estimated using TeSmTL and SBmTL (first derivatives of regression lines in Figure 2 (for model see Table 3). SBmTL, blue line, TeSmTL, green line. In both panels, lines represent the pattern averaged over the sexes.

For comparisons of the age‐dependent trajectories of TeSmTL with SBmTL, both sets of measurements were transformed to a standard normal distribution. When plotted this way (Figure 3, top panel), age‐dependent trajectories of TeSmTL and SBmTL were indistinguishable from each other over the largest part of the age range, with a minor divergence at older age where data density was relatively low (Figure 3, top panel). In absolute values, however, age‐dependent TeSmTL shortening was consistently slower than that SBmTL (Figure 3, bottom panel).

**FIGURE 4:**
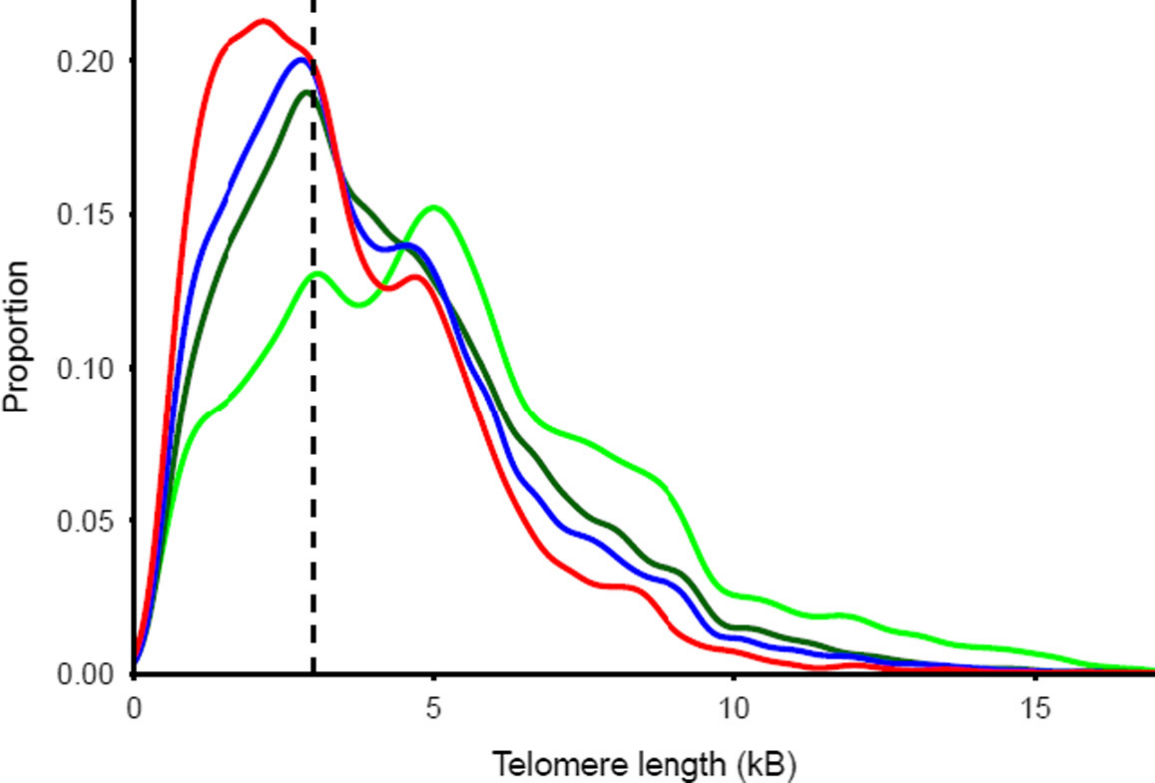
Distribution of TL (kb) within samples measured using TeSLA for different age classes Age 0, green; age 1–25 years, dark green; age 26–65, blue; age 65+, red. *N* = 54,399 telomeres in 334 individual samples. Lines drawn using geomdensity function of the R‐package ggplot2.

TeSLA generates data of single telomeres (162.9 ± 48.0 telomeres/sample in this study), enabling examination of within‐sample single TL variation. Figure 4 displays the single‐telomere TL distribution for four age groups. It shows a shift toward shorter single telomeres and a compression of the distribution with age, as confirmed by a decline with age of the SD of single‐telomere TL, calculated within samples (*t* = 10.22, *n* = 334, *p* < 0.0001).

**FIGURE 5:**
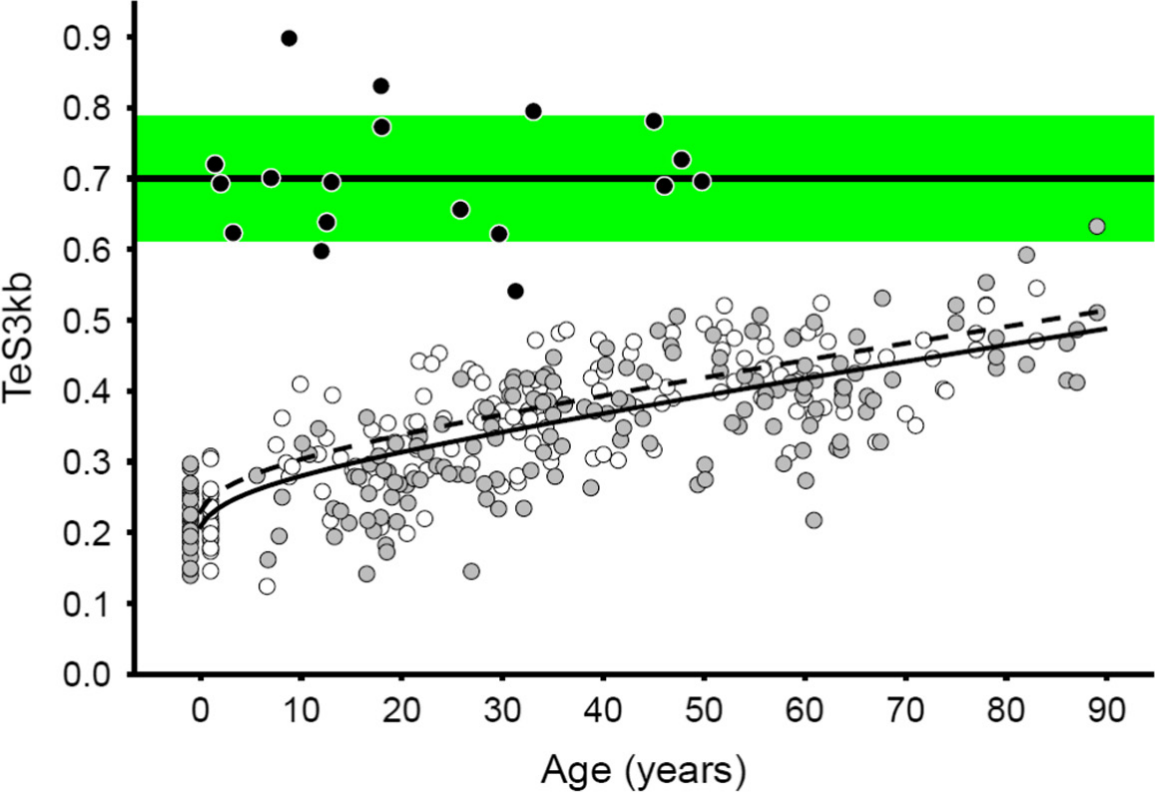
TeS3kb, proportion (%) of telomeres shorter than 3 kb in each DNA sample in relation to age. Females: open markers, continuous line. Males: grey markers, dashed line. Dyskeratosis congenita patients: solid black markers, mean ± 1 SD indicated by the green shaded area. New‐borns slightly displaced left and right of age zero to reduce the number of overlapping data points.

Focusing on the age‐dependent increase in the proportion of telomeres <3 kb (TeS3kb), we found that the TeS3kb increase with age (Figure 5) was best described with the same combination of terms as TeSmTL (Table 3). As per TeSmTL, little overlap was observed between TeS3kb in participants from the general population and DC/TBD patients (illustration of TeSLA for a patient with a DC/TBD disorder vs. a healthy adult of the same age is shown in Figure S2). Through an iterative process (100 bp intervals) we then determined the threshold of TL showing the strongest association with age. Telomeres <4.4 kb showed the strongest age dependency (*R* ^2^ = 0.77; <3 kb, *R* ^2^ = 0.59).

Though each of the three TL metrics (SBmTL, TeSmTL, and TeS3kb) converged to the telomeric brink, defined as the TL parameters observed in DC/TBD patients, very few persons might reach it during their lifespan. To illustrate this point, we extrapolated the observed ageing trajectories to estimate the age at which the telomeric brink would be reached and found this to be ages of 170 and 169 years for SBmTL and TeSmTL, respectively, and age of 185 years for TeS3kb. To evaluate the robustness of this inference, given the uncertainty in estimating the telomeric brink, we also estimated the age at which the telomeric brink plus one SD (or −1 SD for TeS3kb) is reached, and found this to be at ages of 129, 132, and 142 for SBmTL, TeSmTL, and TeS3kb, respectively. We stress, however, that these estimates are based on a relatively small sample and extrapolations over more than 80 years, and they simply serve to illustrate the unlikelihood of reaching the telomeric brink, given the present life expectancy.

### 2.3 The sex effect on mean and short LTL dynamics over the lifespan

**FIGURE 6:**
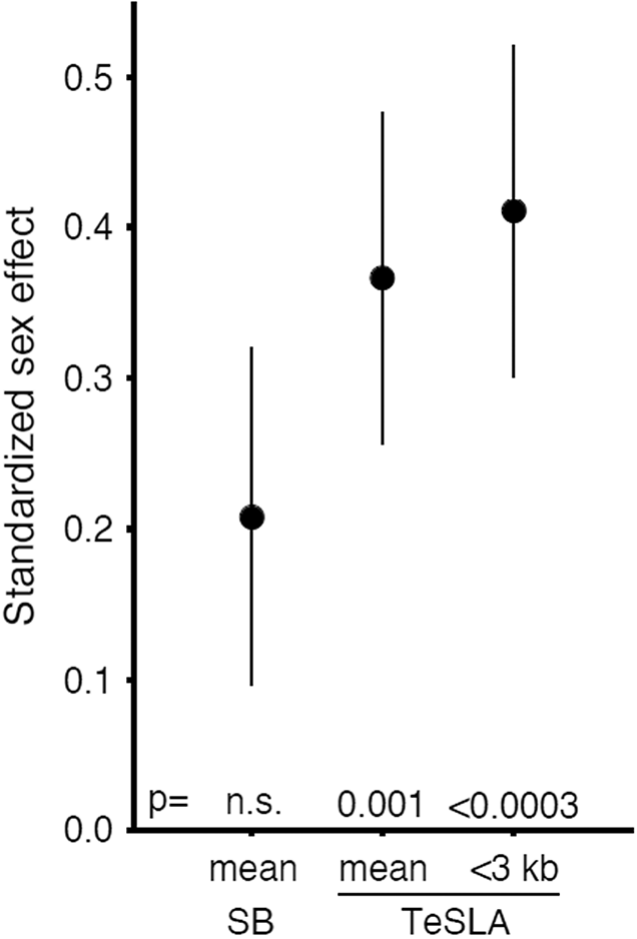
Sex difference in telomere length Estimates (± SE) are for three different telomere metrics, SBmTL, mean TL by SB; TeSmTL mean TL by TeSLA, and TeS3kb proportion of telomeres shorter than 3 kb. All three expressed relative to the pooled standard deviation of that metric calculated over the residuals of the models in Table 2, without sex.

Males displayed a shorter LTL than females **(**Table 3). Statistically, this sex gap was better captured by TeSmTL (*p* = 0.001) compared to SBmTL (*p* = 0.063, Table 3). While numerically, the sex gap differed only slightly between results generated by the two methods, the explained variance by the (identical) complete model was lower for TeSLA based data (Table 3). We illustrate the more pronounced sex effect in the TeSLA‐based data by calculating the standardized difference (Figure 6), which is the sex effect scaled to the residual variation without sex (models in Table 3).

## 3 DISCUSSION

Our knowledge of LTL change with age is fragmented and surprisingly limited (Aubert et al., 2012; Steenstrup et al., 2017). Most population‐based research relies on qPCR measurements, which generate data in relative units, that is, amplified telomere product (T) divided by a single‐gene product (S), without information on absolute TL. T/S data, whose metric differs across laboratories, thus generate little quantitative insight on LTL shortening with age. Moreover, SB and flow‐FISH, which is calibrated by SB, do not usually measure short telomeres among the telomeres in each cell (Kimura, Stone, et al., 2010), and thus overestimate LTL.

We also note that previous studies suggested that the mean value of the X region might be more than 3 kb across individuals (Cawthon, 2002; Hultdin et al., 1998), but our findings do not support this view, because HCs from patients with DC/TBDs display TRFs shorter than 0.5 kb (illustrated in Figure S2); these include the canonical region and the X region. Although we do not know which chromosomes or cells are the source of these ultrashort telomeres, the X region for these telomeres is shorter than 0.5 kb. This means that the trajectories of telomeres <3 kb we show in this work reflect changes with time that principally apply to the canonical component of the telomeres.

We found that the shapes of the trajectories of SBmTL and TeSmTL with age were similar, although in absolute terms SBmTL shortened at a faster rate than TeSmTL. We also found that the buildup of telomeres <3 kb increased with age in tandem with the shortening of SBmTL and TeSmTL. In fact, the upward trajectory of TeS3kb was characterized by the same terms that described the downward trajectory of TeSmTL. More detailed analyses revealed that the proportion of telomeres <4.4 kb attained the highest association with age, and 4.4 kb may thus be the optimal threshold to tally the age‐dependent buildup of short telomeres measured by TeSLA.

However, even in octogenarians the proportions of these short telomeres were less than those observed in patients with DC/TBDs, who usually present with aplastic anemia, suggesting that their HCs reached their replicative limits. Therefore, age‐dependent buildup of these short telomeres is unlikely to severely impede erythropoiesis causing severe anemia during the life course of most individuals in the general population. Our extrapolations of the trajectories of TL parameters in leukocytes support this conjecture, but should be regarded as broad estimates, since the values of the TL parameters depend on the cell compositions of donated DNA samples. In adults, TL is shorter by ~1 kb in lymphocytes than neutrophils (Aubert et al., 2012). In addition, patients with DK/TBDs often present with neutropenia in addition to aplastic anemia (Niewisch et al., 2022).

While the accumulation of HC telomeres <3 kb during the human life course might not severely affect erythropoiesis under steady state conditions, in some adults it can still limit the ability of lymphoid cells, particularly T cells, to undergo clonal expansion in response to infection. The magnitude of this expansion, a model suggests, might be stymied by age‐dependent HC telomere shortening (Anderson et al., 2022). However, such a model is based on LTL, that is, mean TL, data. Except for small studies (Benetos et al., 2021; Lai et al., 2021), no information is available about whether the shortest telomeres in HCs and other somatic cells provide a better insight of TL‐dependent biological processes and health outcomes in humans. Moreover, leukocytes consist of cells of myeloid and lymphoid lineages with different rates of age‐dependent shortening with age (Aubert et al., 2012), whereas our trajectories are based on TL parameters of all these lineages. We note, however, that within an individual, TL variation across these lineages and other somatic cells is much smaller that the inter‐individual TL variation (Aubert et al., 2012; Daniali et al., 2013; Kimura, Gazitt, et al., 2010). Therefore, we anticipate that the broad dynamics of TL shortening and buildup of the shortest telomere we show in this work would apply to specific lineages. That said, as indicated more than a decade ago, the focus on a specific leukocyte lineage largely depends on the investigators' TL‐related hypothesis (Kimura, Gazitt, et al., 2010).

LTL is shorter in males than females from birth onward (Aubert et al., 2012; Factor‐Litvak et al., 2016; Gardner et al., 2014; Hjelmborg et al., 2015; Hunt et al., 2020; Steenstrup et al., 2017), as confirmed in the present study. Statistical support for this sex gap was considerably stronger for TeSmTL and TeS3kb than for SBmTL. This difference cannot be attributed to greater precision of TeSLA compared to SB, because repeatability of the latter method is slightly higher (see Materials and Methods). Instead, the difference might stem from the different restriction enzymes used by the two methods. Enzymes used in TeSLA‐generated shorter TRFs, with less contribution of the X region, which might be variable across persons. Reducing individual variation in this region might explain the higher statistical power in cross‐sectional analyses of TeSLA when investigating effects of sex and perhaps other TL‐related features.

We acknowledge limitations of the study, including the following: Although TeSLA is a reliable method to tally and measure single telomeres, the method does not identify the chromosomal origins of these telomeres. Our study is a composite of diverse participants, originating from different groups in different countries. We have not explored potential health outcomes in participants from the general population since sample sizes for any given age group was too small.

In conclusion, this is the first study showing the buildup of short telomeres in HCs from birth onward over nine decades of human life. TeSmTL and the ability of TeSLA to capture short/ultrashort telomeres provide the most refined picture of LTL dynamics in humans. Based on data in the general population and in patients with DC/TBDs, the study suggests that the buildup of telomeres <3 kb over the lifespan might be insufficient to severely impede erythropoiesis under steady state condition in most healthy individuals as they get older. Therefore, the contribution of HC TL dynamics to aging‐related human diseases and longevity (Codd et al., 2021) might be exerted through other mechanisms, for example, clonal hematopoiesis of indeterminate potential (Aviv & Levy, 2019; Nakao et al., 2022). In addition, the study has generated further insight into the X region, suggesting that it is shorter than that previously estimated.

## 4 MATERIALS AND METHODS

### 4.1 Subjects

LTL parameters were measured in DNA samples from 334 participants from five studies (Table 1). SBmTL data from four studies were published (Benetos et al., 2018, 2019; Factor‐Litvak et al., 2016; Nettle et al., 2021), and one study is ongoing. For the present study, we performed TeSLA on 18 patients with DC/TBDs and had enough DNA to perform SB in 7 of these patients. All TL measurements were performed at the Rutgers' laboratory. Participants in all studies provided written informed consents approved by ethic committees and institutional review boards for the research use of their samples.

### 4.2 LTL measurements

DNA samples for both SB and TeSLA passed an integrity test by resolving 20 ng of DNA on a 1% (w/v) agarose gel.

SB measurements were performed (in duplicate) as previously described (Kimura, Stone, et al., 2010). Briefly, DNA was digested using the Hinf I and Rsa I restriction enzymes (Roche Applied Sciences, Mannheim, Germany). Digested DNA and DNA ladders were resolved on 0.5% agarose gels for 16 h (2 V/cm). We also performed SB measurements de novo in eight DNA samples digested with the restriction enzymes used for TeSLA (below). Precision of SBmTL measurements as indicated by the ICC is 0.98 (Nettle et al., 2021).

TeSLA measurements: These measurements were performed as previously described (Lai et al., 2021). In brief, extracted DNA is ligated at the overhangs of telomeres to single‐stranded adaptors that contain seven nucleotides of telomeric C‐rich repeats at the 3′ end, which is complementary to the G‐rich overhang followed by a unique sequence for PCR. The DNA was then digested with restriction enzymes (BfaI, CviAII, MseI, and NdeI) (New England Biolabs, Ipswich, MA) and subsequently ligated at the proximal end of telomeres and DNA fragments with doubled‐stranded TeSLA adapters with specific primer sequence for PCR. Multiple PCR reactions were then performed to amplify ligated telomeres. PCR products were resolved on a 0.85% agarose gel (1.5 V/cm for 19 h). After gel electrophoresis, Southern blot analysis is applied to detect amplified telomeres. The algorithm of TeSLA detects each telomere band location, annotates the band size, and hence the raw data consist of a series of band sizes for each sample. The TeSLA software displays histogram of the telomere band size distribution and calculates relevant parameters. Precision of TeSmTL measurements as indicated by the ICC is 0.90 (Lai et al., 2022).

### 4.3 Statistics

Using R (R Core Team, 2019) and the lme4 package (Bates et al., 2015), analyses were performed on SBmTL, TeSmTL, TeS3kb, and TeS4kb. TeS3kb and TeS4kb were arcsine‐square‐root‐transformed to meet the homoscedasticity assumption. As LTL shortens at a faster rate in early life, we fitted different combinations of terms, including polynomials (linear, squared, and cubed) and age natural log‐transformed (using age +1 because of newborns' age = 0), and compared the fit to the data using the Akaike information criterion and visual inspection of graphs, including raw data and the model fit. We also fitted the correlations of TL parameters with age using restricted cubed splines, leveraging the R package Hmisc. While the latter approach enables fitting very complex patterns, it has a limited ability to interpret results beyond visual inspection. We used the fitted spline to determine visually equations that reveal a close match between the spline and the equation fitted using a conventional approach. Cohort identity was included as random effect in the analyses since DNA donors came from five cohorts of different ages and countries of residence. Reported *R* ^2^ values refer to variation explained by the fixed effects only. Males have shorter LTL than females and we thus included sex in all analyses. Interactions between age and sex were not significant (not shown).

## AUTHOR CONTRIBUTIONS

Conceptualization: AA. Methodology: T‐PL, SV, SS. Investigation: SS, SG, ST, AB, ES, PF‐L. Visualization: SV. Supervision: AA. Writing—original draft: AA. Writing—review and editing: AA, SV,SS, SG, ST, AB, ES, PF‐L.

## FUNDING INFORMATION

T‐PL's telomere research is supported by the National Institutes of Health U01AG066529 and New Jersey Alliance for Clinical and Translational Science Career Development Award NJACTS KL2 TR003018. SS' and SG's research is funded in part by the intramural research program of the Division of Cancer Epidemiology and Genetics, National Cancer Institute. AB's research is supported by the French National Research Agency (ANR), Translationnelle: N°ID RCB: 2014‐A00298‐39: 2014–2017 and partially supported by the French PIA project “Lorraine Université d'Excellence” reference ANR‐15‐IDEX‐04‐LUE, and the Investments for the Future program under grant agreement no. ANR‐15‐RHU‐0004. ST's is supported by the French PIA project “Lorraine Université d'Excellence” reference ANR‐15‐IDEX‐04‐LUE; FHU project CARTAGE‐PROFILES (Aviesan, 2021–2025); French National Research Agency (ANR), Translationnelle: N°ID RCB: 2014‐A00298‐39. ClinicalTrials.gov Unique Identifiers: NCT02176941(TELARTA) and NCT01391442 (STANISLAS). PF‐L's is funded by the following National Institutes of Health grants: R01 HD071180 from the National Institute of Child Health and Development and R21 ES023582 and 5P30 ES009089 from the National Institute of Environmental Health Sciences. Funded by the National Institutes of Health (NIH). AA's telomere research is supported by the National Institutes of Health (grants R01HD071180 and U01AG066529) and a grant from the Norwegian Research Council (ES562296).

## CONFLICT OF INTEREST STATEMENT

All other authors declare they have no competing interests.

## Supporting information


Figures S1‐S2.

## Data Availability

All data are available in the main text or the supplementary materials.
