# Supplementary material for: Buildup from birth onward of short telomeres in human hematopoietic cells
Source: Aging Cell. 2023 Apr 28;22(6):e13844. doi: 10.1111/acel.13844 (PMC10265151; doi:10.1111/acel.13844)
Supplement: Supplementary file 1 — Figures S1‐S2. [file ACEL-22-e13844-s001.pdf]

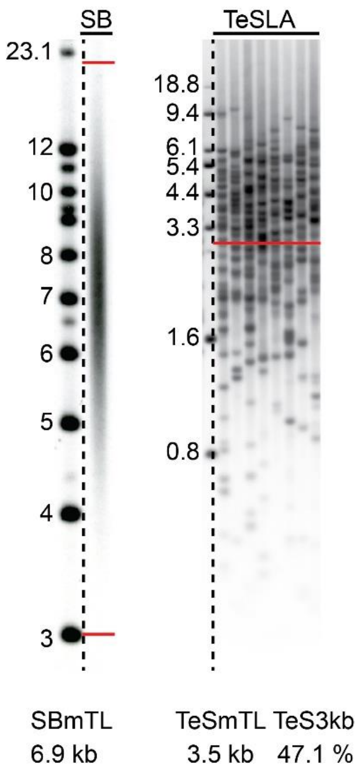

Fig. S1.

Supplemental Figure 1. Illustrations of SB and TeSLA in leukocytes from a 53-year-old healthy man. Molecular weight references are displayed to the left of the vertical interrupted lines. Red horizontal lines denote the range of TL captured by SB (left). The TeSLA output (right) is resolved in multiple lanes, all derived from one DNA sample. Each band represents a single telomere. The TeSLA algorithm enables annotating the band size and calculating relevant parameters. Note the short and ultrashort telomeres < 3 kb (below the horizontal red line). SBmTL, mean TL by SB (kb); TeSmTL, mean TL by TeSLA (kb); TeS3kb, proportion of telomeres shorter than 3 kb by TeSLA (%).

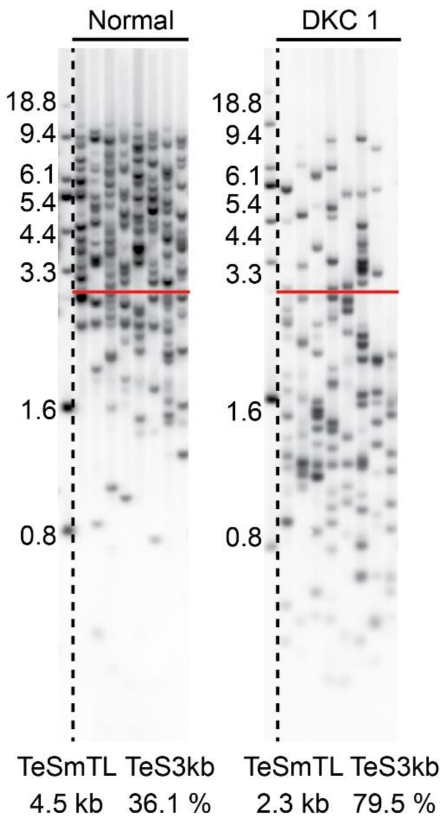

Fig. S2.

Supplemental Figure 2. Illustration of TeSLA in leukocytes from two males, aged 33 years.

Left, healthy subject; right, patient with *DKC1* mutation. 79.5% of telomeres of the patient with dyskeratosis congenita are < 3 kb, while 36.1% of telomeres are < 3 kb in the healthy subject.
